# Older People’s Help-Seeking Behaviors in Rural Contexts: A Systematic Review

**DOI:** 10.3390/ijerph19063233

**Published:** 2022-03-09

**Authors:** Ryuichi Ohta, Yoshinori Ryu, Chiaki Sano

**Affiliations:** 1 Community Care, Unnan City Hospital, Unnan 699-1221, Japan; yoshiyoshiryuryu.hpydys@gmail.com; 2 Department of Community Medicine Management, Faculty of Medicine, Shimane University, Izumo 690-0823, Japan; sanochi@med.shimane-u.ac.jp

**Keywords:** help-seeking, rural, lay care, professional care, quality of care, older people, elderly

## Abstract

Help-seeking behavior (HSB) is vital for older people to sustain their health. As people in aging societies increasingly demand management of their multiple symptoms, communities should encourage HSBs. In rural communities, insufficient healthcare and human resources influence older people’s health. However, no related comprehensive evidence exists so far. This study investigates the present condition of older people’s HSBs in rural contexts in aging societies. We conducted a systematic review by searching six databases (PubMed, Cochrane Library, EMBASE, Medline, and Web of Science) for original studies regarding HSBs of older people in rural contexts published until January 2022. Extracted articles were analyzed based on participants, settings, HSB causes and contents, and older people’s HSB outcomes in rural contexts. Sixteen studies were included in the systematic review: seven investigated the associations between HSBs and participants’ backgrounds, and three the quality of life. Six studies investigated HSB perception, diagnosis, clarifying HSB contents, professional care trend, self-rated health, and mortality. Unlike few studies investigating the association between HSBs and health-related outcomes, this systematic review explains the current evidence regarding rural older people’s HSBs. Due to insufficient evidence from longitudinal studies in clarifying interventions for effective HSBs, future studies should use observational and interventional designs.

## 1. Introduction

Help-seeking behaviors (HSBs) are vital for older people to sustain their health conditions. HSB is defined as any action of energetically seeking help from healthcare services or from trusted people in the community and includes understanding, guidance, treatment, and general support [1]. Individuals take measures to deal with various symptoms based on their judgment. These behaviors are referred to as HSBs, influenced by their preferred type of care for addressing health concerns [1]. HSBs include modifying eating habits, performing regular exercise, consulting, utilizing professional care, and early detection of cancers based on their symptoms [2,3,4]. They are used in contexts where individuals manage their symptoms using lay and professional resources [5]. HSBs are associated with health conditions because they aid in effectively utilizing medical care before disease progression [6,7]. However, their quality may depend on people’s backgrounds, health literacy, and environment.

Aging societies demand that older people manage their multiple symptoms using various resources [8,9]. HSBs in such societies will vary according to the available healthcare resources. In urban areas, where sufficient medical resources are available, people can choose among various medical and healthcare professionals [10]. However, this situation can also be associated with the inappropriate use of medical care [10]. Older people may use professional care for their mild symptoms without balanced consideration of their need for medical care. In turn, the inappropriate usage of medical care may not nurture peoples’ knowledge or skills regarding managing their mild symptoms. In addition, lack of knowledge and skills to handle symptoms can cause delay to access to medical care in cases of critical disease.

Furthermore, lack of information resources and poor accessibility of medical care knowledge and sites can deter HSBs, especially among rural older people. Populations in rural areas lack adequate medical care resources. When they experience symptoms, they must judge symptom severity and choose among considerably limited medical resources and accessibility of medical care. However, older people may lack knowledge on medical-related issues because of insufficient resources [11]. Although the Internet and social media have made knowledge-gathering easier, they are primarily used by young and middle-aged individuals, at a rate of more than 60% [12,13,14,15]. Older people, in contrast, tend to obtain information from television and newspapers, and have Internet use of less than 50% [12,13,14,15]. Insufficient information may lead to underuse of professional care, especially for critical symptoms. The progression of symptoms could then cause various diseases, leading to clinical problems such as multimorbidity and polypharmacy [16,17]. Inadequate information and healthcare provision to older people living in rural areas may also affect their HSBs [18,19].

The lack of healthcare and human resources impinges on older people’s health conditions in rural communities [20,21]. Communities need to modify HSBs to effectively manage the older people’s health conditions in rural areas [22,23]. Clarification of the present comprehensive evidence regarding the relationship between older people’s HSBs in rural areas and related factors and health outcomes is essential to design interventions for improving their health conditions. However, there is no systematic review of older people’s HSBs in rural contexts. Various HSBs, mixed with health-seeking behaviors, can enhance everyday health condition, such as modifying eating habits, performing regular exercise, and undergoing screening. This time, we focused on HSBs for symptoms among older people, because the clarification of the relation of behaviors to symptoms could contribute to deep consideration of provision of healthcare and revision of HSBs. Thus, the purpose of this study is to analyze the present condition of older people’s HSBs in response to symptoms in rural contexts.

## 2. Methods

### 2.1. Study Design and Search Strategy

We conducted a systematic review, following the Preferred Reporting Items for Systematic Reviews and Meta-Analyses (PRISMA) statement [24]. We used four databases (PubMed, Cochrane Library, EMBASE, and Web of Science) to search for original studies regarding older people’s HSBs in rural contexts published till January 2022. The search strategy was based on the following title/abstract keywords in English: 1. older people or the elderly; 2. help-seeking behavior; and 3. rural. The reference lists of relevant studies were also reviewed to identify those missed in the database search.

### 2.2. Inclusion and Exclusion Criteria

Literature searches and data extraction were independently conducted by three investigators (RO, YR, and CS), and any discrepancies were resolved through discussion. In this study, databases were searched for original studies regarding older people’s HSBs in rural contexts. Studies conducted without clear descriptions of the aim, participants, or outcomes were excluded. Details of the inclusion and exclusion criteria are presented in Table 1. 

### 2.3. Data Extraction

One of the investigators (RO) extracted data from each original study using a purpose-designed data extraction form. The other two investigators (YR and CS) checked the extracted data. Extracted data were categorized into participants, purpose, research methodology, countries, causes of HSB, contents of HSB, and outcomes regarding HSB. We also examined two further categories: quality of life (QOL), which is defined as individuals’ perception of their position in life in the context of the culture and value systems in which they live and in relation to their goals, expectations, standards, and concerns, and self-rated health (SRH), which is defined as the health people feel subjectively [7,23].

### 2.4. Analysis

To ensure that the study quality was reliable, we collected and analyzed the data based on the PRISMA standards. This systematic review was registered in PROSPERO (registration number: 307215) and conducted according to the procedure. The contents of participants, purpose, study methodology, countries, causes of HSB, contents of HSB, and outcomes regarding HSB were descriptively analyzed. The setting was described as the name of the country. With regard to the contents of HSBs, they were briefly categorized as lay care, professional care, and lay and professional care based on a previous study [25]. Lay care refers to the care provided to care receivers by individuals without specialized training. Professional care entails care provided by trained, paid professionals such as physicians, nurses, and pharmacists. To describe the cause of HSBs, concrete diagnoses or categorizations such as general symptoms (symptoms not specified), acute symptoms, chronic diseases (symptoms from chronic diseases), and psychiatric disorders (symptoms from psychiatric disorders) were used. Regarding the outcomes of the studies, we used the following categories: perception of HSB (from qualitative research); the trend of professional care (how frequently professional care was used); clarifying the contents of HSB (what kinds of HSBs were used for symptoms); associations with backgrounds; mortality; QOL; and SRH.

## 3. Results

### 3.1. Search Results

**Figure 1:**
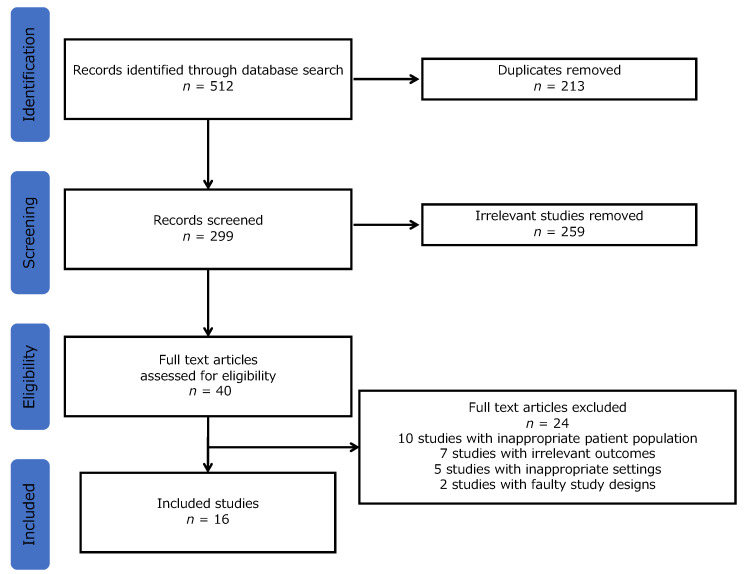
Flowchart of study selection.

By searching the databases following the protocol, 512 studies were identified. A total of 472 studies were excluded because of duplication and irrelevance. Through the review of complete texts, 24 studies were excluded for the following reasons: 10 studies with inappropriate patient population, 7 studies with irrelevant outcomes, 5 studies with inappropriate settings, and 2 studies with faulty study designs (Figure 1). Finally, sixteen studies were included in this systematic review (Table 2).

### 3.2. Study Characteristics

The number of studies is categorized as follows: two studies were performed in the United States, three in China, four in Japan, and three in India. One study each was performed in Vietnam, Poland, Nigeria, and Australia. Of them, 12 were cross-sectional studies, 2 used a cohort study, and 1 study each used qualitative and mixed-methods designs (Table 3).

### 3.3. The Causes of HSBs, Contents of HSBs, and Study’s Outcomes

We found that four studies each investigated general symptoms and acute symptoms. Three studies investigated chronic symptoms, and two explored depression. Further, one study each investigated general anxiety disorders, psychiatric diseases, and myocardial infarction.

Regarding the contents of HSBs, twelve studies investigated professional care, two lay care, and two studies investigated both professional and lay care.

Concerning the outcomes of the studies, seven studies investigated the associations between HSBs and participants’ backgrounds; three studies examined the QOL; and one study each investigated the perception of HSBs, diagnosis, clarifying contents of HSB, the trend for professional care, SRH, and mortality (Table 4).

### 3.4. Rural Older Patients’ HSBs and Outcomes

#### 3.4.1. Associations with Backgrounds

In the review of the included studies, educational intervention, gender, educational level, socioeconomic status, past medical histories, personal brief, mistrust of mental health providers, stigma, health status, alcohol consumption, utilization of family practice, and living with families were associated HSBs among older people living in rural areas [26,27,30,33,34,36,38]. Education was found to be essential for HSBs. Higher education was associated with higher usage of professional care for symptoms of depression [27]. Lower education levels were associated with a higher likelihood of untreated morbidity due to chronic diseases [34]. Regarding educational interventions, educational workshops for people with psychiatric disorders increased the use of professional care [26].

#### 3.4.2. Barriers

Gender affected HSBs in various ways. Being female was associated with low usage of professional care for symptoms of depression [27]. In addition, female patients had a lower probability of seeking treatment for other psychiatric disorders [36]. Lower educational level, monthly income, and the presence of one or more major medical conditions were associated with lower usage of professional care for the symptoms of depression [27]. Low socioeconomic status also impinged on effective HSB. It was observed that low socioeconomic status acted as a barrier to treating psychiatric disorders [30]. Low-income households were associated with low treatment-seeking for chronic diseases [38]. Health conditions also reduced the use of self-care. Good health conditions and no recent alcohol consumption were both associated with self-treatment for symptoms [33]. Older adults with low socioeconomic status had a lower probability of seeking treatment for psychiatric disorders [36]. The most reported barrier to treatment for depression was the personal belief that “I should not need help”. Other commonly reported barriers included mistrust of mental health providers, thinking treatment would not help, stigma, and not being willing to talk to a stranger about private matters [30]. Familiarity with medical care and support from families positively affected HSBs. Familiarity with medicine was associated with self-treatment [33]. Older people living with a spouse, as compared to those living alone, had a lower likelihood of having untreated morbidities [34].

#### 3.4.3. QOL, Diagnosis, and Perception of HSBs

HSB is associated with QOL. People with higher QOL were less likely to use inpatient services [31]. Familiarity with healthcare was associated with high QOL [32]. In addition, HSB with a trend of self-management for mild symptoms was related to a high QOL [37]. One cross-sectional study showed that the three most common causes of geriatric emergencies were acute malaria, hypertensive crises syndrome, and acute hypertensive heart failure in rural districts in a developing country [29]. A qualitative study showed that older people living in rural areas believed that receiving help in late life was a reward for a good life. In addition, this population believed that by understanding and following the rules of help-seeking among older people, formal helpers might more efficiently and effectively meet their needs [25].

#### 3.4.4. Clarifying the Content of HSBs

A mixed-method study showed the concrete contents of HSBs for mild symptoms among older people living in rural areas. HSBs for mild symptoms were categorized into two groups: lay care and professional care. The following HSBs were categorized as lay care: do nothing; self-management (e.g., changing lifestyles, sleeping, resting, and taking a bath); seeking information; consulting family, friends, and community members; using complementary or home medicine; and buying over-the-counter drugs. The following HSBs were classified as professional care: consulting pharmacists or primary care physicians, visiting medical institutions (other than primary care physicians), and visiting emergency rooms of the general hospital (including calling for an ambulance). The most common HSB associated with mild symptoms was consultation with primary care physicians, followed by self-care and home medicine. The test–retest reliability for mild symptoms was 0.836 for lay care and 0.808 for professional care [35].

#### 3.4.5. Trend for Professional Care, SRH, and Mortality

A cross-sectional study regarding HSBs with symptoms related to myocardial infarction showed that 76.2% would call an ambulance in response to chest pain. Merely, 80% were able to provide emergency phone numbers. Among respondents who declared they would not call an ambulance, 38.7% were afraid of in-hospital COVID-19 infection or healthcare system collapse [39]. Another cross-sectional study showed an association between HSBs and SRH. It demonstrated that using both lay and professional care was significantly associated with high SRH [40]. One cohort study showed a relationship between HSB and mortality. It indicated that mortality was higher in rural than in urban women, and rural women made fewer visits to general practitioners and medical specialists compared to their urban counterparts [28].

## 4. Discussion

This systematic review clarified the current evidence regarding HSBs among older people living in rural areas. Most studies have focused on the association between HSBs among older people living in rural areas and their backgrounds and socioeconomic status. However, few have investigated the association between HSBs of older people and their health conditions using subjective health scaling. The present study not only focused on the continual investigation of associations but also the relationships between HSBs and health conditions that would aid in designing interventions for older people living in rural areas.

One of the factors affecting HSBs was the lack of healthcare resources [30,33,34], which in rural contexts can lead to a false sense of improvement in subjective health. Studies have indicated that few primary care facilities and emergency medicines may lead to deterioration in people’s health conditions [9,41,42]. Due to inadequate healthcare resources, individuals may be compelled to modify their original HSBs [43]. Changes in their HSBs can cause mental stress, distress, and poor health conditions [4]. Careless interventions for HSB can also lead to a deterioration in health conditions. Therefore, a balance between healthcare resources and individual healthcare needs should be considered while designing healthcare interventions to facilitate the effective use of medical care.

Another related factor affecting HSBs was knowledge of HSBs and diseases; the knowledge should be provided by respecting culture in communities. This review showed that educational levels and education on HSBs may be significant for better care. Therefore, knowledge of health management among older rural populations is crucial. As older people’s information on healthcare resources regarding health management in rural areas can be limited [5,44], health management information should be provided via various resources [45,46]. Less exposure to information about health management may impinge on effective self-management and professional care use [46]. Ineffective self-management and low use of professional care may lead to poor health conditions [8,9]. Contrarily, efficient usage of primary care and emergency medicine could be related to people’s better perceptions regarding their health [8,9,47,48,49]. Therefore, effective knowledge provision is essential for better subjective health.

Supporting rural older people’s HSBs is crucial for improved healthcare. This review revealed that the relationship between HSBs and living with families and the community may be related to the efficient usage of professional care. Understanding health management and promoting effective self-management accompanied by other HSBs can be provided in communities [50,51]. In rural areas with few healthcare resources and knowledge, older people have to manage their symptoms by themselves [9,21,52]. The provision of essential knowledge for health management and access to professional care by families and community members can help in the management of health conditions and symptoms among older individuals in rural contexts [53,54]. Family and community members should drive such education.

Clarification of the relationship between HSBs and health-related outcomes can be critical for designing interventions in rural contexts. Preferences for care are affected by health conditions and healthcare resources concerning the provision of interventions for HSBs. However, this review showed a lack of evidence of the effectiveness of interventions for HSBs. One should focus on HSB modifications among older rural populations. Regarding interventions for HSB modification, the differences between urban and rural healthcare situations should be considered in different contexts.

Supporting rural older people’s knowledge regarding health management using both lay and professional support is essential. As this review indicated high consultation rates with primary care physicians, primary care facilities should impart guidance and informational resources for self-management for older adults’ mild symptoms. Additionally, effective collaboration among community members for information provisioning regarding health management could be useful for inculcating better health conditions in rural areas [9,55]. Rural communities are aging rapidly and losing their previous active relationships because young people are settling outside and cultural amalgamation is occurring in rural areas [9,21,52]. Therefore, mutual knowledge sharing about health management should be promoted to improve health management in older people [55].

Personal factors in communities, such as privacy and isolation, should be respected while designing interventions. The low rate of consultations with community members can be related to privacy issues that may render rural individuals reluctant to confess their difficulties to others, causing isolation in communities [9]. Isolation can be associated with low self-management use [9]. Considering HSBs, lay and professional care resources respecting rural individuals’ privacy and isolation need to be established [9,56]. Engagement and empowerment of people are essential to get help in difficult situations and facilitate HSBs. A helping attitude should be cultivated by all community members [53,54].

To enhance rural older adults’ understanding of and self-efficacy/intention regarding effective lay and professional care use, continuous dialogues among older adults, community members, and healthcare professionals are required [57]. Through these dialogues, older adults can better understand healthcare conditions in communities, assess their knowledge regarding HSBs, and use healthcare resources [9,20]. They can become empowered and motivated to learn with the help of healthcare professionals [9]. This in turn can contribute to establishing systems and education regarding HSBs according to context, leading to better health. As this review contains studies from several countries facing aging problems, further investigation is be needed regarding HSBs among older people and the relationship with the health conditions. Thus, future research should build upon this review to assess the needs of rural older populations for professional care and their balance with healthcare capacity in rural contexts, to promote lay care and effective use of professional care.

One limitation of this study is the definition of help-seeking. The definition has been clarified in a previous study, but HSBs can mean different things to different researchers [10,58]. Therefore, misclassification may have occurred. Moreover, during the review, we intensively examined the included studies employing the term “help-seeking” [10]. Subsequent studies can investigate other keywords such as “health-seeking”, “healthcare-seeking”, and “illness behaviors”. Another limitation is the lack of a meta-analysis in this systematic review, which was not possible due to the variety of study designs, participants, outcomes, and study settings. Next, the reviewers are Japanese and specialize in public health and HSBs, both factors that may have introduced bias in the analysis and synthesis of results. Although search engines used worldwide were used, the researchers might have missed including some studies. In addition, one future spin-off alongside this traditional systematic review might be to undertake a meta-ethnography of existing qualitative studies of older rural people and health behaviors/experiences/attitudes, looking for HSB themes.

## 5. Conclusions

This systematic review has clarified the association and relationship between HSBs and their health conditions among older people living in rural areas. However, there is a lack of evidence regarding diagnosis and trend for professional care regarding HSBs and also regarding the relationship between HSBs and subjective and objective health in rural contexts over long periods of time, measured through longitudinal studies. To gain better clarity regarding the nature of interventions for HSBs, future studies should investigate the relationships using observational and interventional studies.

## Figures and Tables

**Table 1:** Inclusion and exclusion criteria.

Criteria	Inclusion	Exclusion
**Population**	Rural older people/the rural elderly	Other age groups
**Setting**	Rural areas	Not rural areas
**Types of study**	Qualitative, quantitative, mixed-methods	Non-empirical studies (editorial, news)
**Outcome**	Outcomes associated with HSBs	Outcomes not associated with HSBs
**Other**	Abstract available Full text available in English	Abstract not available Full text not available in English

**Table 2:** Studies reporting rural older individuals’ HSBs.

Study	Country	Study Design	Participants	Causes of HSB	HSB	Outcome	Results
Jett 2002, [26]	United States	Qualitative research	*n* = 41; M and F	General symptoms	Professional care	Perception of HSB	Older individuals believe that receiving help in late life is a reward for a good life. By understanding and following the rules of help-seeking, formal helpers might more efficiently and effectively meet their needs.
Sakamoto 2004, [27]	Japan	Cross-sectional study	*n* = 285; M and F	Depression	Professional care	Associations with backgrounds	Participation in mental health workshops was associated with frequent consultation with professional care.
Ma 2008, [28]	China	Cohort	*n* = 127; M and F	Depression	Professional care	Associations with backgrounds	Female sex, lower educational level, monthly income, and the presence of one or more major medical conditions were associated with lower use of professional care with the symptoms of depression.
Vagenas 2009, [29]	Australia	Cohort	*n* = 12,778; F	General symptoms	Professional care	Mortality	Mortality was higher in rural than in urban women. Rural women reported fewer visits to general practitioners and medical specialists.
Iloh 2012, [30]	Nigeria	Cross-sectional	*n* = 216; M and F	Acute symptoms	Professional care	Diagnosis	The three most common causes of geriatric emergencies were acute malaria (33.8%), hypertensive crises syndrome (19.0%), and acute hypertensive heart failure (18.1%).
Brenes 2015, [31]	United States	Cross-sectional	*n* = 478; M and F	General anxiety disorder	Professional care	Associations with backgrounds	The most reported barrier to treatment was the personal belief that “I should not need help”. Other commonly reported barriers included practical barriers (cost, not knowing where to go, distance), mistrust of mental health providers, not thinking treatment would help, stigma, and not wanting to talk with a stranger about private matters.
Pham 2018, [32]	Vietnam	Cross-sectional	*n* = 523; M and F	General symptoms	Professional care	QOL	People with higher QOL were less likely to use inpatient services.
Zhang 2019, [33]	China	Cross-sectional	*n* = 31,464; M and F	Chronic diseases	Professional care	QOL	One-year and two-week access to healthcare was found to be associated with QOL scores at the 10th and 90th quantiles, respectively. Access to healthcare affects the self-assessed health and QOL of the elderly.
Xu 2020, [34]	China	Cross-sectional	*n* = 216; M and F	General symptoms	Lay care	Associations with backgrounds	The factors associated with self-treatment were better health status, no recent alcohol consumption, and no utilization of family practice.
Srivastava 2020, [35]	India	Cross-sectional	*n* = 9973; M and F	Chronic diseases	Professional care	Associations with backgrounds	Older individuals living with a spouse in comparison to those living alone had a lower likelihood to have untreated morbidities. Additionally, the elderly from rural areas and having lower levels of education had a higher likelihood of untreated morbidity.
Ohta 2021, [25]	Japan	Mixed-method	*n* = 267; M and F	Acute symptoms	Lay and professional care	Clarifying contents of HSB	The most common behavior with mild symptoms was consulting with primary care physicians, followed by self-care and using home medicine. The test–retest reliability for mild symptoms revealed kappa values of 0.836 for lay care and 0.808 for professional care.
Srivastava 2021, [36]	India	Cross-sectional	*n* = 31,464; M and F	Psychiatric disorders	Professional care	Associations with backgrounds	Older adults, who were females and with a lower socioeconomic background had a lower probability of seeking treatment for a psychiatric disorder.
Ohta 2021, [37]	Japan	Cross-sectional	*n* = 1066; M and F	Acute symptoms	Lay care	QOL	The HSBs with a trend of using self-management were related to a high QOL.
Chauhan 2021, [38]	India	Cross-sectional	*n* = 31,464; M and F	Chronic diseases	Professional care	Associations with backgrounds	Treatment-seeking is relatively low among the elderly in low-income households.
Korman 2021, [39]	Poland	Cross-sectional	*n* = 194; M and F	Myocardial infarction	Professional care	Trend for professional care	76.2% would call an ambulance in response to chest pain. Merely 80% were able to recall the emergency phone number. Among respondents who declared they would not call an ambulance, 38.7% were afraid of in-hospital COVID-19 infection or healthcare system collapse.
Ohta 2021, [40]	Japan	Cross-sectional	*n* = 169; M and F	Acute symptoms	Lay and professional care	Self-rated health	Using both lay and professional care was significantly associated with high self-rated health.

Footnotes. HSB: help-seeking behavior; F—female; M—male; QOL—quality of life.

**Table 3:** Distribution of reviewed studies by countries and research design.

Variable	Number of Studies	Percentage
Countries		
United States	2	12.5%
China	3	18.8%
Japan	4	25%
India	3	18.8%
Vietnam	1	6.25%
Poland	1	6.25%
Nigeria	1	6.25%
Australia	1	6.25%
**Study design**		
Cross-sectional	12	75%
Cohort	2	12.5%
Qualitative	1	6.25%
Mixed-method	1	6.25%

**Table 4:** The causes of HSBs, the contents of HSBs, and the study’s outcomes.

Variable	Number of Studies	Percentage
Cause of HSB		
General symptoms	4	25%
Acute symptoms	4	25%
Chronic diseases	3	18.8%
Depression	2	12.5%
General anxiety disorder	1	6.25%
Psychiatric diseases	1	6.25%
Myocardial infarction	1	6.25%
**Content of HSBs**		
Professional care	12	75%
Lay care	2	12.5%
Lay and professional care	2	12.5%
**Study outcomes**		
Associations with backgrounds	7	43.8%
Quality of life	3	18.8%
Perception of HSB	1	6.25%
Diagnosis	1	6.25%
Clarifying contents of HSB	1	6.25%
Trend for professional care	1	6.25%
Self-rated health	1	6.25%
Mortality	1	6.25%

Note. HSB: help-seeking behavior.

## Data Availability

All relevant datasets used in this study are presented in the manuscript.
